# Supplementary material for: Machine translationese of large language models: Dependency triplets, text classification, and SHAP analysis
Source: PLoS One. 2026 Jan 9;21(1):e0339769. doi: 10.1371/journal.pone.0339769 (PMC12788636; doi:10.1371/journal.pone.0339769)
Supplement: Attachment — Submitted filename: Response to Reviewers.docx [file pone.0339769.s002.docx]

**Manuscript ID:** PONE-D-25-08735
**Title:** Machine Translationese of Large Language Models: Dependency Triplets, Text

Classification, and SHAP Analysis
**Authors:** Shukang Zhang; Chaoyong Zhao

Dear Editor and Reviewers

We sincerely appreciate your time and constructive feedback. Below is our point-by-point response to all comments.

****Response to Academic Editor****

1. **Style Requirements**: We have carefully reviewed the manuscript and believe it now aligns with PLOS ONE’s formatting guidelines, including file naming conventions. The text has been adjusted to follow the provided style template to the best of our knowledge. Please let us know if any further modifications are needed.
2. **Data/Code Sharing**: The data and author-generated code has been uploaded to GitHub https://github.com/KiemaG5/LLM-translationese.

### ****Response to Reviewer 1****

****Comment**:** “Add pseudocode for the proposed approach.”
****Response**:**
We appreciate the suggestion and have added detailed pseudocode as in Fig 2 to clarify the methodology. The pseudocode outlines:

1. **Dependency Parsing**: Extraction of triplets
2. **Feature Engineering**: Vectorization of triplet frequencies .
3. **Model Training**: Cross-validation workflow.
4. **SHAP Interpretation**: Global, local and dependence analysis of discriminative features
5. **Feature Reduction:** Iterative pruning of low-importance features while monitoring F1-score to assess robustness

### ****Response to Reviewer 2****

#### ****Comment 1**:** “Framing the Methodology as Deep Learning”

****Response**:**
We appreciate the suggestion and have revised terminology to avoid overstatement:

1. **Rep**laced “deep learning” with “neural network-based approach”.

#### ****Comment 2**:** *Hyperparameter Tuning and Overfitting”*

****Response**:**
We appreciate the reviewer’s insightful suggestion regarding model optimization. However, our primary goal was to validate the general utility of features, not model optimization. Thus:

1. Expanded**Methodology** to include **16 classifiers** (SVM, MLP, CatBoost, etc.) from scikit-learn and other libraries, all using **default parameters** to demonstrate feature robustness without hyperparameter tuning.
2. Top F1 Score on Test Set: **SVM (93%)**, MLP (92%), CatBoost (91%), significantly above random (50%). Lower performers (e.g., Decision Tree: 74%, Naïve Bayes: 79%) still surpassed chance, confirming feature discriminability.

#### ****Comment 3**:** “Unclear Data Split”

****Response**:**

We appreciate the suggestion, and have clarified in Sections Text classification and Model performance

1. **10-fold cross-validation** on the full dataset (776 samples).

#### ****Comment 4**:** “Missing Simple Baselines”

****Response**:**
We appreciate this constructive suggestion and have added:

1. **16 models including Decision Tree (74%), Naïve Bayes (79%)** and K-Nearest Neighbors (81%) as baselines.
2. **SHAP analysis** focused on **SVM, MLP, CatBoost & Decision Tree, Naïve Bayes** K-Nearest Neighbors (top and last 3 performing models) to identify the similarity and dissimilarity in globally impactful features.

We believe these revisions address the concerns accordingly. Thank you again for your valuable input.

Sincerely,
Shukang Zhang
East China Normal University
mikeashjd@163.com
