# Supplementary material for: Machine translationese of large language models: Dependency triplets, text classification, and SHAP analysis
Source: PLoS One. 2026 Jan 9;21(1):e0339769. doi: 10.1371/journal.pone.0339769 (PMC12788636; doi:10.1371/journal.pone.0339769)
Supplement: Attachment — Submitted filename: Response_to_Reviewers_auresp_2.docx [file pone.0339769.s003.docx]

To: The Editor

Manuscript ID: PONE-D-25-08735R1

Title: Machine Translationese of Large Language Models: Dependency Triplets, Text Classification, and SHAP Analysis

Dear Editor,

Thank you for providing us with the opportunity to revise our manuscript. We are grateful to the reviewers for their time and insightful feedback. We have carefully considered all comments and have revised the manuscript accordingly.

Below are our point-by-point responses to the reviewers’ comments.

**Response to Reviewer #1**

We sincerely thank Reviewer #1 for their positive assessment and are pleased that they found our revisions satisfactory.

**Response to Reviewer #2**

We thank Reviewer #2 for their detailed and rigorous feedback. We have addressed the three main concerns as follows:

1. On the comparison with deep models: We appreciate the suggestion to compare with models like BERT and Llama. The primary focus of our paper is to evaluate the power of interpretable, linguistic features (dependency triplets) in distinguishing translation types. This feature-engineering approach is fundamentally different from end-to-end deep learning models, which operate on embeddings and are less directly interpretable. We have clarified this scope in the manuscript and acknowledged that a direct comparison with fine-tuned deep models is a valuable direction for future research.

2. On hyperparameter tuning and overfitting: We thank the reviewer for raising this important point. Our decision to use default parameters for the 16 classifiers was deliberate. Our central hypothesis was that the proposed features are inherently discriminative. By demonstrating strong performance (e.g., F1-score of 0.92 with MLP) across a wide range of models without specific tuning, we provide robust evidence for the general utility of our features. We believe this is a stronger testament to the features’ power than optimizing a single model. We have added a clear explanation of this rationale in the Methodology section.

3. On the data split and SHAP analysis: We thank the reviewer for this insightful comment. We agree that a held-out test set is a gold standard. We chose k-fold cross-validation as it provides a more robust and less biased estimate of generalization performance than a single random split.

Regarding the SHAP analysis, our goal was to explain what patterns the models learned during this robust CV process. Our methodology for calculating SHAP values aligns with standard examples in the official SHAP documentation. To address the valid concern about explaining overfit patterns, we highlight that our findings are based on the consistent high performance across 16 different classifiers. The fact that our features work well across such a diverse set of models strongly suggests that the patterns identified by SHAP are genuinely discriminative and not artifacts of a single, potentially overfit model. We have clarified our methodology and reasoning in the revised manuscript. We also acknowledge that a three-way split or nested CV is a highly rigorous standard, which we will certainly consider for future extensions of this work.

**Response to Reviewer #3**

We sincerely thank Reviewer #3 for their encouraging and constructive feedback. We have addressed all minor suggestions in the revised manuscript:

1. A thorough language check has been performed to improve clarity.

2. We have added a discussion of the dataset’s limitations (single book and language pair) in the Discussion and Conclusion sections.

3. We have included a rationale in the Methodology section for using default classifier parameters.

4. All figures, especially the SHAP plots, have been regenerated in higher resolution for better clarity.

We believe the manuscript has been substantially improved with these revisions. We hope you now find it suitable for publication.

Sincerely,
Shukang Zhang
East China Normal University
mikeashjd@163.com
